# Editorial: Insights in cardiac rhythmology 2021

**DOI:** 10.3389/fcvm.2022.1003843

**Published:** 2022-08-16

**Authors:** Andrea Ballatore, Gaetano Maria De Ferrari, Matteo Anselmino

**Affiliations:** Division of Cardiology, Department of Medical Sciences, “Città della Salute e della Scienza di Torino” Hospital, University of Turin, Turin, Italy

**Keywords:** arrhythmias, electrophysiology, innovation, cardiac, atrial fibrillation

2021 has been a year of transition and, again, of great change. Old but never quiescent challenges have risen anew after the pandemic, being the effects of climate change and international political crisis blatant these days. The scientific community has proven an unprecedented sense of cooperation and showed its strength by providing a safe and effective solution to one of the greatest health and social crises of the last century. The widespread availability of vaccines for COVID-19 allowed a gradual relaxation of restraint measures and a progressive return to normality.

Indeed, 2021 scientific production has been characterized by this sense of international corporate effort, being the scientific community aware of its crucial role in providing effective solutions; cardiac rhythmology included (Figure 1).

**Figure 1:**
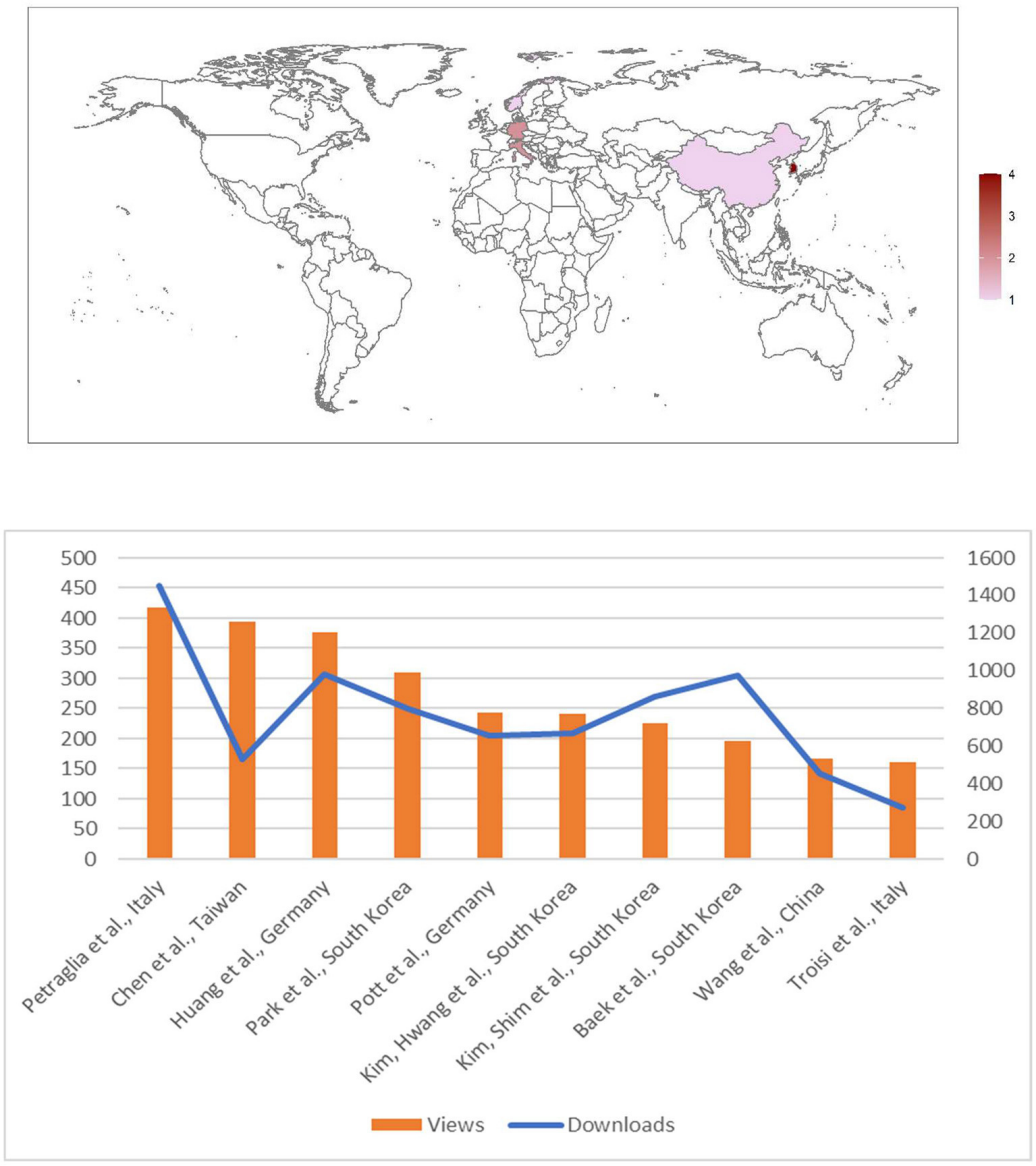
Metrics (lower panel; update on July 18th, 2022) and number of articles per Country of Authors' origin (upper panel) of papers published in Frontiers in Cardiovascular Medicine: Insights in cardiac rhythmology 2021.

A great consideration has been given to atrial fibrillation (AF) and its therapeutic strategies. Out of the classic questions, attention to the metabolic relationships of the arrhythmia has emerged. New evidence shows how epicardial adipose tissue is linked to perioperative AF following cardiac surgery (Petraglia et al.); this relation is mediated by an increased level of inflammation pointing toward the importance of immunology and metabolic interplay in the development of the arrhythmia. Moreover, Kim, Shim et al. demonstrated that malnutrition, which is associated with an increased risk of AF occurrence, is also linked to a greater risk of complications after AF catheter ablation. Similarly, a low BMI and female gender was associated to a greater risk of non-transient phrenic nerve palsy (Pott et al.); in this case, it is probably due to an increased proximity of the right phrenic nerve to the ablation target in these patients, however, as suggested by the authors, the increased levels of epicardial adipose tissue in men and in those with higher BMI may play a relevant role in thermal insulation. Therefore, bearing in mind the recent experience with gliflozines, widening their effects from diabetes mellitus treatment to cardiovascular prevention in heart failure patients, greater attention should be raised to metabolic aspects when approaching AF patients.

Technological improvement is constant and new tools are available on almost a daily basis in cardiac electrophysiology, allowing, for instance, the development of new ablation strategies and a deeper knowledge of AF pathophysiology (Kim, Huang et al.). In this respect, pulmonary vein isolation as the sole ablation target in persistent AF is currently questioned and a more comprehensive approach may be advocated (1). The development of new mapping technologies will lead to a further reduction of fluoroscopy use and greater diffusion of “zero X-rays” procedures (Baek et al.; Troisi et al.; Wang et al.) also for complex and extensive-substrate transcatheter procedures.

The attention to new treatment strategies for AF management is rooted in the results of the EAST-AFNET 4 trial, which showed the benefit of rhythm control strategies in the early phases of the disease (2). The publication of the exciting 1 year follow-up results of pulse-field ablation trials suggests that this technology will soon have an increasingly relevant role in the invasive management of arrhythmias (3). Growing evidence point toward catheter ablation as first line treatment (4); however National Health services could struggle to cope with this high demand of ablation procedures, and efforts should be aimed to increase their availability.

In addition, the approach to the treatment of ventricular tachycardia is undergoing a complete change with catheter ablation assuming a major role. The increasing knowledge of arrhythmogenesis and improved substrate definition pave the way for a more effective management for this dire arrhythmia (Huang et al.). Eventually, artificial intelligence will play a progressively central role in medicine, with the use of large database allowing to develop new algorithms in order to better stratify patients and predict response to therapy and disease progression (Chen et al.; Park et al.).

However, many questions still lay unsolved. A clear definition of the relationship between AF and stroke and firm evidence on need and timing of anticoagulation for subclinical AF episodes are still lacking. The solution of these conundrums is at quest of several studies. Given these premises, we foresee that future years will be at least as scientifically exciting as 2021, and we eagerly await apprises that will soon arrive; so, keep in touch!

## Author contributions

MA and AB conceived the editorial. MA and GD revised the text. All authors contributed to the article and approved the submitted version.

## Conflict of interest

MA has received educational grants from Abbott, is consultant for Biosense Webster and proctor for Medtronic. The remaining authors declare that the research was conducted in the absence of any commercial or financial relationships that could be construed as a potential conflict of interest.

## Publisher's note

All claims expressed in this article are solely those of the authors and do not necessarily represent those of their affiliated organizations, or those of the publisher, the editors and the reviewers. Any product that may be evaluated in this article, or claim that may be made by its manufacturer, is not guaranteed or endorsed by the publisher.

## References

[1] Saglietto A Ballatore A Gaita F Scaglione M Ponti R De Ferrari GM De. Comparative efficacy and safety of different catheter ablation strategies for persistent atrial fibrillation: a network meta-analysis of randomized clinical trials. Eur Heart J Qual Care Clin Outcomes. (2021) 2021:qcab066. 10.1093/ehjqcco/qcab066 34498687

[2] Kirchhof P Camm AJ Goette A Brandes A Eckardt L Elvan A. Early rhythm-control therapy in patients with atrial fibrillation. N Engl J Med. (2020) 383:1305–16. 10.1056/NEJMoa2019422 32865375

[3] Reddy VY Dukkipati SR Neuzil P Anic A Petru J Funasako M. Pulsed field ablation of paroxysmal atrial fibrillation: 1-year outcomes of IMPULSE, PEFCAT, and PEFCAT II. Clin Electrophysiol. (2021) 7:614–27. 10.1016/j.jacep.2021.02.014 33933412

[4] Saglietto A Gaita F De Ponti R De Ferrari GM Anselmino M. Catheter ablation vs. anti-arrhythmic drugs as first-line treatment in symptomatic paroxysmal atrial fibrillation: a systematic review and meta-analysis of randomized clinical trials. Front Cardiovasc Med. (2021) 8:664647. 10.3389/fcvm.2021.664647 34095254 PMC8175669

